# Clinicopathological significance of primitive phenotypes in early gastric cancer with differentiated histology

**DOI:** 10.1186/s13000-021-01128-w

**Published:** 2021-07-31

**Authors:** Zhi-Yi Zhou, Jie Sun, Qing Guo, Hai-Bin Zhao, Zhi-Hua Zhou

**Affiliations:** 1 Department of Pathology, The Affiliated Wuxi People’s Hospital of Nanjing Medical University, Wuxi, Jiangsu Province China; 2 Center of Clinical Research, The Affiliated Wuxi People’s Hospital of Nanjing Medical University, Wuxi, Jiangsu Province China; 3 Department of Pathology, The 904 Hospital of Joint Logistic Support Force of People’s Liberation Army, North Xinyuan Road 101, Wuxi, 214044 Jiangsu Province China

**Keywords:** Early gastric cancer, Primitive phenotype, SALL4, GPC3, Endoscopic resection

## Abstract

**Background:**

Certain gastric cancers exhibit some primitive phenotypes, which may indicate a high malignancy. In histologically differentiated early gastric cancer (EGC), the presence and the clinicopathological significance of the primitive phenotype remain unclear.

**Methods:**

Using immunohistochemical staining we detected the expression of three primitive phenotypic markers SALL4, Glypican-3(GPC3), and AFP in whole tissue sections of differentiated EGC (gastrectomy specimens, *n* = 302). For those cases with primitive phenotypes, we analyzed their clinicopathological features and evaluated whether the criteria for endoscopic resection were met.

**Results:**

We found that 9.3% (28/302) of all differentiated EGC cases have primitive phenotypes, and most of these cases (25/28) exhibit a histomorphology similar to conventional differentiated EGC. Patients with primitive phenotypes had a deeper invasion, a higher rate of ulcer and lymphatic invasion than cases without primitive phenotype. Moreover, patients with primitive phenotypes displayed a significantly higher frequency of LNM than those without (57.1% vs 8.8%, *P* < 0.001). Multivariate analysis revealed that presence of primitive phenotypes was an independent risk factor for LNM (*P* = 0.001, HR 6.977, 95% CI: 2.199–22.138). Interestingly, we found 2 cases with primitive phenotypes developed LNM, and they both met the expanded indications of endoscopic resection for differentiated EGC.

**Conclusions:**

A small number of differentiated EGC have primitive phenotypes, which were closely related to LNM and were an independent risk factor for LNM. Given its highly aggressive behavior, differentiated EGC with primitive phenotypes should be evaluated with stricter criteria before endoscopic resection, or considered to give an additional surgical operation after endoscopic resection.

## Introduction

Early gastric cancer (EGC) refers to gastric cancer that does not invade deeper than the submucosa, regardless of lymph node metastasis (LNM). Nowadays, endoscopic resection, including endoscopic mucosal resection and endoscopic submucosal dissection, has become the standard treatment for EGC [1]. The effects of endoscopic resection are equivalent to that of surgical operation, and it improves the patient’s life quality, avoiding possible complications caused by gastrectomy [2–4].

Endoscopic resection of EGC is applied based on the premise that the patients do not have LNM, and it is vital to assess the risks of LNM for EGC. At present, indications for endoscopic resection have been established, and these indications can evaluate LNM risks of EGC. Several clinicopathological variables, such as tumor size, depth of invasion, ulcer presence, lymphatic/vascular invasion and tumor differentiation are included in these indications. As to tumor differentiation, gastric cancer can be classified as the differentiated or undifferentiated based on its histologic appearance: if cancer cells form a tubular or papillary structure, it is classified as differentiated gastric cancer; if gastric cancer consists of signet ring cell carcinoma or mucinous adenocarcinoma, it is classified as undifferentiated gastric cancer [5]. Most of EGC were differentiated EGC. Although differentiated EGC has a lower incidence of LNM than undifferentiated EGC, it is still important to evaluate LNM risks for differentiated EGC.

Recently, accumulating evidence indicated that certain gastric adenocarcinomas have some primitive phenotypes, and this subtype of gastric cancer displays a high malignancy [6, 7]. A study by Yamazawa et al. revealed that among 386 cases of gastric adenocarcinoma, 93 had primitive phenotypes, and the primitive phenotype was embodied in the expression of the embryonic stem cell markers (SALL4 and CLDN6) and the oncofetal proteins AFP and Glypican-3(GPC3) by cancer cells [6]. Notably, gastric cancer with primitive phenotypes was very aggressive and prone to develop LNM and remote metastasis, leading to a very poor prognosis [6]. Moreover, some rare subtypes of gastric adenocarcinoma, such as hepatoid adenocarcinoma, yolk sac tumor, and gastric adenocarcinoma with enteroblast differentiation (GAED) also display primitive phenotypes, expressing abovementioned primitive markers [8–12].

For gastric cancer with primitive phenotypes, histomorphology may not reflect the true degree of differentiation, and GAED is a representative example: it is histologically similar to conventional differentiated gastric adenocarcinoma with a tubular or papillary structure, and GAED ought to be classified as differentiated gastric cancer according to its tubular or papillary histology. However, GAED displays the phenotypes of immature differentiation, and primitive phenotypic markers (SALL4, GPC3, and AFP) are frequently expressed in GAED [12–14]. The risks of LNM and remote metastasis in GAED are very high, and the outcomes of GAED are poor [12–14], exhibiting a high malignancy similar to undifferentiated gastric cancer. Therefore, in histologically differentiated gastric cancer, primitive phenotypes may be present, and the existence of primitive phenotype is associated with an aggressive behavior. Nevertheless, in histologically differentiated EGC, the presence and the clinicopathological significance of the primitive phenotypes remain unclear.

## Methods and materials

### Patients

This study included 302 cases of differentiated EGC that underwent surgical resection from 2008 to 2018 in our hospital. All specimens and patient information were obtained with institutional review board approval. Postoperative pathological examinations confirmed that all cases were differentiated EGC. Averagely, 16.9 lymph nodes were retrieved from each case (range: 2–38).

EGC is defined that cancer cells invade not deeper than the submucosal layer. T1a gastric cancer means tumor invades the lamina propria and or muscularis mucosae, while T1b gastric cancer means tumor invades into the submucosal layer. For T1b, if the tumor invasion frontier is less than 500 μm from the mucosal muscle layer, it is classified as SM1, and if it is greater than or equal to 500 μm, it is classified as SM2. The identification of differentiated gastric cancer is based on criteria previously published [5]: cancer cells form tubular or (and) papillary structures, lacking solid nests or single scattered cancer cells, and mucinous adenocarcinomas are excluded. Clinical and pathological information of all cases, including age, gender, tumor site, tumor size, depth of invasion, LNM, and vessel invasion were reviewed and recorded.

### Immunohistochemical staining

The archived paraffin blocks from all 302 cases were re-sectioned with a thickness of 4 μm, and immunohistochemical staining was performed on the whole tissue sections. The sections were deparaffinized and hydrated, and then antigen retrieval was performed (95 °C, 15 min). After quenching endogenous peroxidase activity, sections were incubated with primary antibodies for 12 h at 4 °C. Thereafter, sections were incubated with an anti-mouse polymer kit (Envision kit; Beijing Zhongshan Jinqiao Biotechnology Co., Ltd.) for 30 min at room temperature. Then slides were visualized with DAB and counterstained with hematoxylin. Primary antibodies included: mouse anti-human SALL4 antibody (Beijing Zhongshan Jinqiao Biotechnology Co., Ltd., 1:100), mouse anti-human GPC3 antibody (Beijing Zhongshan Jinqiao Biotechnology Co., Ltd., 1:100), mouse anti-human AFP antibody (Beijing Zhongshan Jinqiao Biotechnology Co., Ltd., 1:100), and mouse anti-human D2–40 antibody (Beijing Zhongshan Jinqiao Biotechnology Co., Ltd., 1:100).

Appropriate positive and negative controls were included for each run of immunohistochemical staining, and incubation of samples with antibody dilution buffer without the primary antibodies, was employed as the negative staining control. The positive staining control for the SALL4 antibody, were carcinoma tissues from gastric hepatoid adenocarcinoma, which had been demonstrated strongly positive for SALL4. The positive control for AFP and GPC3 antibodies, were 2 cases of poorly differentiated hepatocellular carcinoma with strong expression of AFP and GPC3.

The stained sections were evaluated by two senior pathologists. When there was a disagreement, they review the sections together and discussed to reach an agreement. A positive result was confirmed if SALL4 expression was localized in the nucleus, AFP was localized in the cytoplasm, and GPC3 and D2–40 were localized in the cell membrane and cytoplasm. When measuring the expression intensity of the markers SALL4, GPC3, and AFP, a 3-point scoring system was used [6]: negative, no cancer cells expressed; focally positive, 1–49% of cancer cells are positive; diffusely positive, ≥50% of cancer cells are positive. Diffuse or focal expression of any one of the three primitive markers was regarded as positive for primitive phenotype. D2–40 is a marker for lymphatic vessels, and cancer cells were confirmed to have lymphatic invasion if D2–40-positive cells surrounded the lumen [15].

### PAS and D-PAS staining

For PAS staining, sections of archived paraffin block (thickness 4 μm) were deparaffinized and placed in water. A solution of 1% periodic acid was added for 10 min, and then sections were incubated with Schiff solution for 15 min for staining. For D-PAS staining, sections were incubated with amylase at 37 °C for 15 min, and then PAS staining was performed as described above.

### Indications for endoscopic resection

According to previous literature [5, 16], indications for endoscopic resection are defined as follows: 1. Absolute indications: intramucosal differentiated adenocarcinoma, no ulcer, tumor size ≤2 cm; 2. Expanded indications, divided into four cases: Ex-1: Intramucosal differentiated adenocarcinoma, without lymphatic vessel invasion (LVI), with ulcers, tumor size ≤3 cm; Ex-2: Intramucosal differentiated adenocarcinoma, without lymphovascular invasion (LVI), no ulcer, no limit on tumor size; Ex-3: undifferentiated adenocarcinoma in the mucosa, no lymphatic invasion (LVI), tumor size ≤2 cm; and Ex-SM1: submucosa differentiated adenocarcinoma, tumor size ≤3 cm, invasion depth ≤ 500 μm.

### Statistics

Categorical variables were compared using chi-square or Fisher exact test, and agreement between two variables was evaluated with Kappa consistency test. Univariate and multivariate logistic regression were used to analyze the risk factors associated with LNM. The statistical software was SPSS 22.0, and *P* < 0.05 was considered statistically significant.

## Results

### Detection of the primitive phenotype in differentiated EGC

**Fig. 1:**
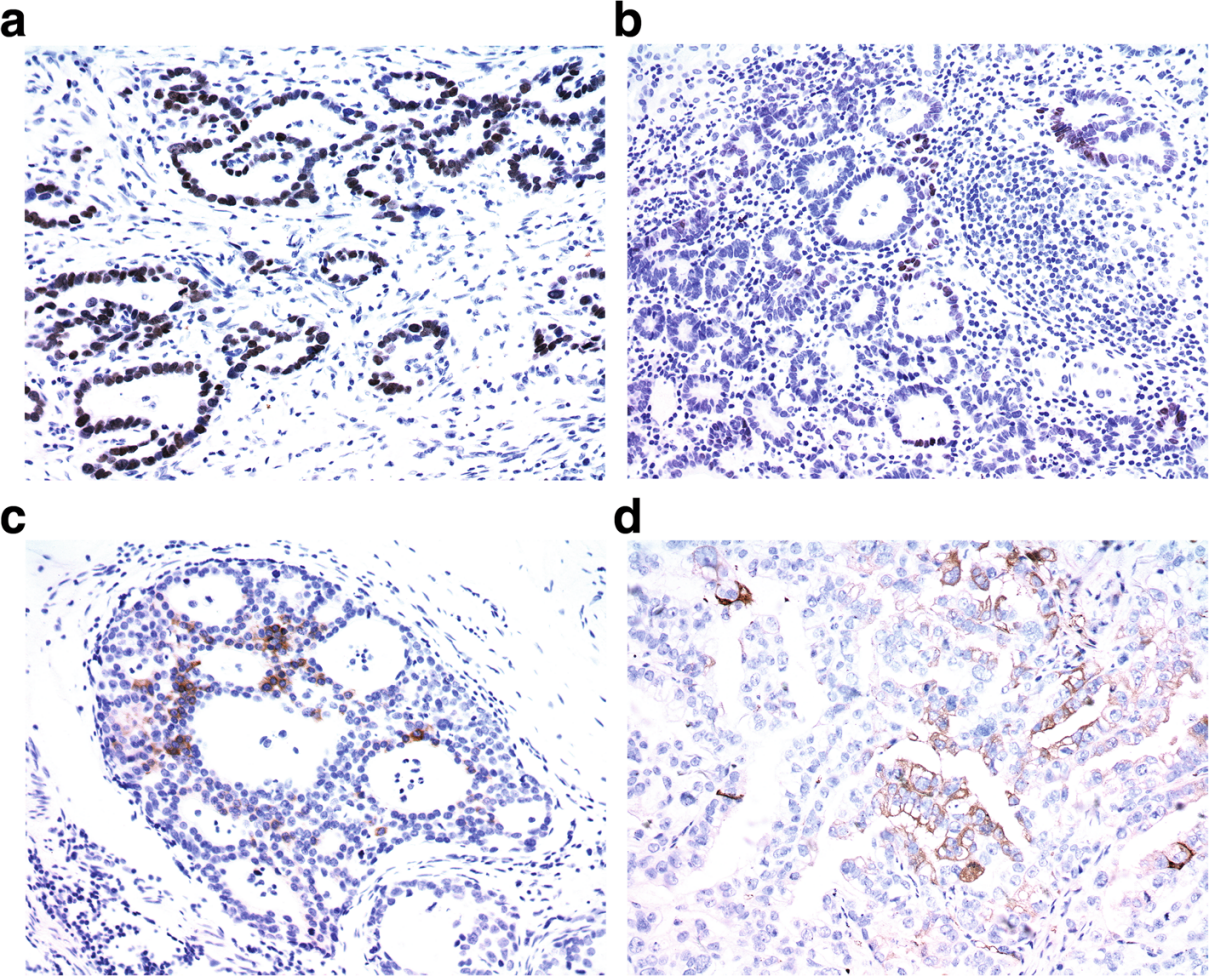
Immunohistochemical staining to assess the expression of primitive phenotypic markers in differentiated early gastric cancer. **A** One representative case displaying diffuse expression of SALL4. **B** Focal expression of SALL4. **C** Focal expression of GPC3. **D** Focal expression of AFP

Immunohistochemistry in whole tissue sections reveal that among 302 cases of differentiated EGC: 1) 24 cases were SALL4 positive, of which 13 were diffusely positive and 11 were focally positive; 2) 8 and 2 were positive for GPC3 and AFP respectively, and both GPC3 and AFP were focally positive (Fig. 1). 3) 274 cases were negative for SALL4, GPC3, and AFP. The expression of primitive markers was shown in Table 1. In total, 28 patients had primitive phenotypes, accounting for approximately 9.3% (28/302) of all cases.

**Table 1:** The expression of the primitive marker in differentiated early gastric cancer with primitive phenotype

Primitive phenotype	Number *n* = 302
SALL4- diffuse	9
SALL4-focal	11
GPC3	4
GPC3 + SALL4-diffuse	2
AFP + SALL4-diffuse	1
GPC3 + AFP + SALL4-diffuse	1
Negative	274

According to Table 1, we found that 2 AFP-positive cases were diffusely positive for SALL4, and one of them also expressed GPC3 focally. The expression of AFP was associated with diffuse expression of SALL4 (*P* < 0.001, Kappa value 0.258), and there also existed an association between AFP expression and GPC3 expression (*P* < 0.001, Kappa value 0.214). Of the 8 patients who were GPC3-positive, 3 were SALL4-positive (2 were diffusely positive, and 1 was focally positive), and GPC3 expression was related to SALL4-diffuse expression (*P* < 0.001, Kappa value 0.278).

### Clinicopathological characteristics of differentiated gastric cancer with primitive phenotype

**Fig. 2:**
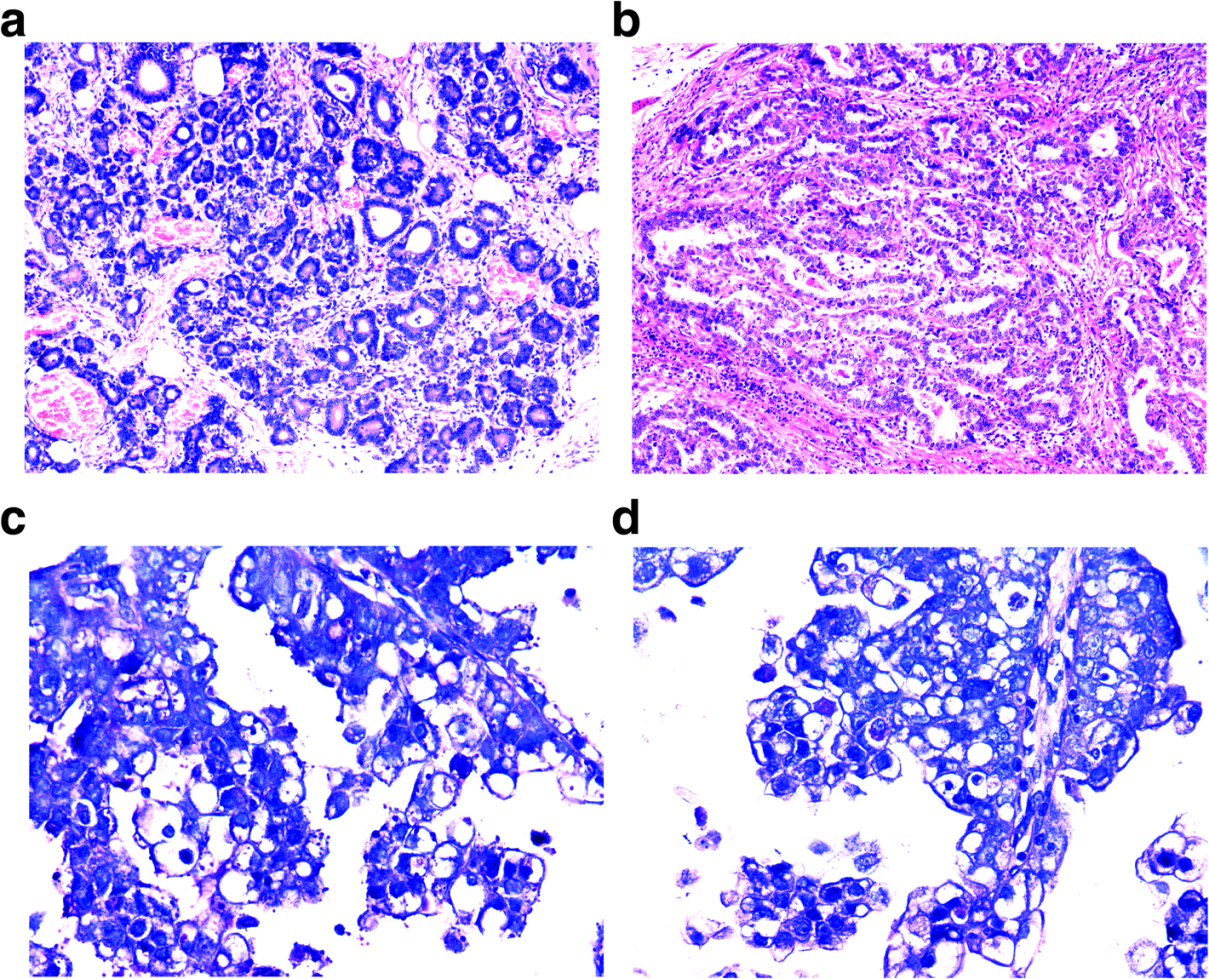
Histological morphology of differentiated early gastric cancer with primitive phenotype. **A** Most of these cases (25/28) have no characteristic morphology, and cancer cells exhibit moderate to marked dysplasia. **B** A few cases (3/28) were characterized by cancer cells with clear cytoplasm, which was morphologically similar to primitive gut epithelium. **C** PAS staining displayed red particles in cancer cells with clear cytoplasm. **D** The red particles in clear cells disappeared when treated with diastase (DPAS staining), demonstrating glycogen deposition in these clear cells

Morphologically, all the 28 cases with primitive phenotypes displayed a tubular, cribriform, or papillary morphology, and the nuclei of the cancer cells were moderately atypical (Fig. 2a). Meanwhile, we found 3 of the 28 cases were GAED. These 3 cases possessed cancer cells with clear or vacuolar cytoplasm (Fig. 2b), and PAS staining and D-PAS staining indicated the presence of glycogen in the clear or vacuolar cytoplasm (Fig. 2c-d). Except for the 3 cases of GAED, the remaining 25 cases with primitive phenotypes lacked recognizable morphological features distinct from conventional differentiated gastric cancer.

Table 2 lists the relationships between primitive phenotypes and clinicopathological variables. Presence of primitive phenotype was not associated with patients’ gender, age, or tumor size. Compared with cases without a primitive phenotype, patients with primitive phenotypes showed a higher rate of ulcer and a deeper invasion, and lymphatic invasion and LNM were more frequent in these cases.

**Table 2:** Relationship between primitive phenotype and clinicopathological characteristics of differentiated early gastric cancer

	Primitive phenotype		***P*** value
	Present *n* = 28	Absent *n* = 274	
**Sex**			0.922
Female	5	51	
Male	23	223	
**Age**			0.257
< 60	4	65	
≥ 60	24	209	
**Location**			0.065
Upper	7	118	
Middle	10	52	
Lower	11	104	
**Ulcer**			0.037
Present	14	84	
Absent	14	190	
**Size**			0.717
≤ 3	22	223	
> 3	6	51	
**Depth**			0.001
T1a	2	117	
SM1	6	42	
SM2	20	115	
**Vessel invasion**			< 0.001
Present	14	32	
Absent	14	242	
**LNM**			< 0.001
Present	16	24	
Absent	12	250	

### Presence of the primitive phenotype was an independent risk factor of LNM in differentiated EGC

As evaluating the risks of LNM is vital for EGC treatment, we further screened clinopathological variables related to LNM and assess the role of primitive phenotypes in predicting LNM. Univariate analysis revealed that gender, age, and ulcer presence was not related to LNM. For cases with primitive phenotypes, 16 patients had LNM, and the incidence of LNM was 57.1% (16/28), which was significantly higher than that of those without primitive phenotypes (8.8%, 24/274). As LNM were present in 40 cases of all the 302 patients, we can deduce that 40% (16/40) of differentiated EGCs with LNM have primitive phenotypes.

In terms of invasion depth, there was no significant difference in LNM rates between SM1 (T1b, < 500 μm) and SM2 (T1b, ≥500 μm) (*P* = 0.530), and LNM rates were higher in these two groups than in T1a. Therefore, they were combined into one group (T1b group). In terms of tumor site, we found that tumors located in the upper stomach had significantly lower LNM rates than tumors located in the middle and lower stomachs. There was no significant difference in the rates of LNM in cases located in the middle and lower stomachs (*P* = 0.554), so these cases were also combined into one group. We also found that tumor size and lymphatic involvement were also closely related to LNM. Table 3 lists these results.

**Table 3:** Relationship between clinicopathological characteristics and lymph node metastasis in differentiated early gastric cancer

	LNM		HR	95% CI	***P*** value
	present *n* = 40	absent *n* = 262			
**Sex**					0.7991
Female	8	48	1		
Male	32	214	0.897	0.389–2.069	
**Age**					0.453
< 60	11	58	1		
≥ 60	29	204	0.750	0.353–1.591	
**Location**					0.001
Upper	5	120	1		
Middle	14	48	7.000	2.390–20.501	
Lower	21	94	5.362	1.949–14.750	
**Ulcer**					0.275
Absent	24	180	1		
Present	16	82	1.463	0.738–2.901	
**Size**					0.008
≤ 3	27	223	1		
> 3	13	39	2.753	1.308–5.793	
**Depth**					< 0.001
T1a	4	115	1		
SM1	11	37	8.547	2.567–28.459	
SM2	25	110	6.534	2.202–19.383	
**Vessel invasion**					< 0.001
Absent	12	244	1		
Present	28	18	31.630	13.812–72.431	
**Primitive phenotype**					< 0.001
Absent	24	250	1		
Present	16	12	13.889	5.891–33.746	

According to the univariate analysis, five factors (primitive phenotype, tumor site, tumor size, depth of invasion, and lymphatic invasion) were closely related to LNM, these factors were further included for the multivariate analysis. As a result, we found that presence of the primitive phenotype (*P* = 0.001, HR 6.977, 95% CI: 2.199–22.138), tumor located in the middle and lower stomach (*P* = 0.008, HR 4.473, 95% CI: 1.487–13.460), and LVI (*P* < 0.001, HR 18.540, 95% CI: 7.273–47.261) were independent high risk factors for LNM. Therefore, presence of the primitive phenotype was a useful marker to predict LNM risks for differentiated EGC.

### Analyzing whether differentiated EGC meets indications of endoscopic resection and the presence of LNM

Next, we investigated whether the 302 cases met the indications of endoscopic resection and the occurrence of LNM. Of the 28 cases with primitive phenotypes, we found that: 22 cases (78.6%, 22/28) did not meet the endoscopic resection criteria (absolute and expanded indications); 2 cases met the absolute indications and had no LNM; 4 cases met the expanded indications for differentiated EGC (Ex-SM1). Interestingly, 2 of the above mentioned 4 cases had LNM. Of the 274 cases without primitive phenotypes, 132 cases (48.2%, 132/274) did not meet the indications for endoscopic resection; 57 met the absolute indications and none of them had LNM; 85 met the expanded indicationsfor differentiated EGC and 1 of them had LNM. As a result, the proportion of patients who did not meet the indications for endoscopic resection was significantly higher in patients with primitive phenotypes than in those without (78.6% vs. 48.2%, *P* = 0.002). Moreover, when the expanded indications was met, the cases with primitive phenotypes were more prone to develop LNM than those without (2/4 vs. 1/85, *P* = 0.005). Table 4 lists the clinicopathological characteristics of patients with LNM who met the expanded indications for endoscopic resection.

**Table 4:** Clinicopathological characteristics of 3 patients with lymph node metastasis that meet the expanded indications for endoscopic resection

Case	Primitive phenotypes	Sex	Age	T stage	Positive nodes	Tumor size	Vessel invasion	Ulcer	Location
1	Present (focal GPC3 +)	M	63	SM1	1	2.8*2.5	0	0	antrum
2	Present (diffuse SALL4+)	M	69	SM1	1	0.8*0.5	0	0	body
3	Absent	M	73	SM1	2	2.0*1.8	0	0	cardia

As shown in Table 4, two cases with primitive phenotypes met the expanded indications for endoscopic resection (Ex-SM1), and both of them had LNM, with an invasion depth of T1b (SM1). However, if the expanded indications for undifferentiated gastric cancer (Ex-3: limited to the mucosa, size ≤2 cm, no vascular invasion) is applied, neither of these 2 cases would conform to these indications. Therefore, when differentiated EGC with primitive phenotypes was regarded as undifferentiated EGC, endoscopic resection may not be applied and we can avoid clinical risks caused by underestimating the incidence of LNM. Alternatively, if primitive phenotypes were found in endoscopically resected specimens, an additional surgical operation might be considered because of high risks of LNM.

## Discussion

To our knowledge, our present study was the first to explore the presence and clincopathological significance of primitive phenotypes in differentiated EGC, and this study provides a valuable indicator for predicting LNM risks in differentiated EGC. The primitive phenotype may be regarded as a marker of undifferentiation. However, we found the primitive phenotypes present in 9.3% (28/302) of histologically differentiated EGC, and cases with primitive phenotypes had a significantly higher frequency of LNM than those without (57.1% vs. 8.8%). Furthermore, our results revealed that presence of the primitive phenotype was an independent high risk factor for LNM in differentiated EGC.

As the presence of primitive phenotype was closely related to LNM, it is necessary to determine the primitive phenotype in differentiated EGC. We found that most of the differentiated EGC with primitive phenotypes lacked recognizable histological features, and only 3 of the 28 cases with primitive phenotypes had characteristic morphology (cancer cells that are clear or vacuole-like) consistent with GAED, while the remaining 25 cases were morphologically difficult to distinguish from conventional differentiated EGC. Therefore, histologic morphology cannot indicate the presence of primitive phenotypes, and it should be determined through immunohistochemical assay of SALL4, GPC3, and AFP. Meanwhile, we found the expression of SALL4 and GPC3 are closely related to that of AFP, and all the AFP-positive cases were also SALL4-positive, which is consistent with previous studies [6, 11]. As all AFP-positive cases simultaneously express SALL4, only detecting the expression of SALL4 and GPC3 might be sufficient to determine the primitive phenotype, which is more economical and more efficient.

As shown in Table 1, we found that of 28 differentiated EGCs with primitive phenotypes, of which 13 were diffusely positive for SALL4, and the remaining 15 only focally expressed GPC3 or focally expressed SALL4. The samples detected in our study were surgical specimens, and if endoscopic biopsy specimens are used for testing, some cases with focal expression of SALL4 or GPC3 may be missed due to the small size of the specimen. Therefore, even if the primitive phenotype is not detected in endoscopic biopsy specimens before endoscopic resection, larger specimens obtained from endoscopic resection should be taken to detect primitive phenotypes for a second time, ensuring that the cases with high risk of LNM not be omitted.

At present, the indications of endoscopic resection for EGC proposed by the Japanese Gastric Cancer Association are crucial for screening patients suitable for endoscopic resection. Many studies have proven that the indications can minimize the risk of LNM. LNM rates are extremely low in those who meet absolute endoscopic resection criteria [16, 17], and it can even be as low as 0 [18]. The LNM rates in those who meet extended indication Ex-1 and Ex-2 are less than 1% [16]; however, in patients who meet the expanded indication Ex-SM1, the LNM rate may increase to 3% [19]. As the relative high risk of LNM for EGC that meets Ex-SM1, it is particularly important to assess the LNM risk prior to endoscopic treatment in these patients. In our study, LNM occurred in 3 patients who met the expanded indications (Ex-SM1) for endoscopic resection. Among these 3 patients, 2 displayed primitive phenotypes. Therefore, when evaluating LNM risks of T1b EGC, detecting the primitive phenotype may be substantially helpful to the present expanded indications of endoscopic resection. Moreover, if the primitive phenotype was found in specimens after endoscopic resection, an additional surgical operation might be applied to eliminate possible LNM.

In our cohort, differentiated EGC with the primitive phenotypes was significantly associated with a deeper invasion and an increased rate of LNM, indicating its highly aggressive behavior and high- grade malignancy. Although differentiated EGC with primitive phenotypes exhibit a well differentiated or moderately differentiated histological morphology, it seems reasonable to classify these cases as undifferentiated adenocarcinoma considering its high malignancy, aggressive behavior, as well as the expression of the primitive markers. If differentiated EGC with the primitive phenotypes were classified as undifferentiated gastric cancer, and assessed with the expanded indications for undifferentiated EGC (Ex-3, undifferentiated cancer, intramucosal, ≤2 cm, no ulcers and vascular invasion), then the abovementioned 2 cases with LNM would not meet Ex-3, and the clinical risk that omitting LNM undiscovered and untreated after endoscopic resection would be avoided.

In summary, we found a few differentiated EGC have primitive phenotypes, which were closely related to LNM. Given its highly aggressive behavior, differentiated EGC with primitive phenotypes should be evaluated with stricter criteria before endoscopic resection, or considered to give an additional surgical operation after endoscopic resection.

## Data Availability

The datasets used and/or analyzed during the current study are available from the corresponding author on reasonable request.

## Abbreviations

EGC: Early gastric cancer
LNM: Lymph node metastasis
LVI: Lymphovascular invasion
